# Supplementary material for: Bearing defect detection based on the improved YOLOv5 algorithm
Source: PLoS One. 2024 Oct 28;19(10):e0310007. doi: 10.1371/journal.pone.0310007 (PMC11515976; doi:10.1371/journal.pone.0310007)
Supplement: Attachment — Submitted filename: Response to Reviewers.pdf [file pone.0310007.s001.pdf]

AUTHORS'RESPONSE TO EDITORS AND REVIEWERS

Dear Editors and Reviewers,

Thank you very much for your kindly comments on our manuscript. There is no doubt that these comments are valuable and very helpful for revising and improving our manuscript. We are pleased to submit the revised research article “Bearing defect detection based on the improved YOLOv5 algorithm”. On the following pages, we present our point-by-point responses to the comments.

We would like also to thank you for allowing us to resubmit a revised copy of the manuscript. We appreciate your time and look forward to your response.

Sincerely,
Peigang Jiao,Ph.D. (corresponding author)
jiaopeigang@163.com

Comments from Academic Editor

Thank you for submitting your manuscript to PLOS ONE. After careful consideration, we feel that it has merit but does not fully meet PLOS ONE's publication criteria as it currently stands. Therefore, we invite you to submit a revised version of the manuscript that addresses the points raised during the review process.

Please submit your revised manuscript by Aug 01 2024 11:59PM. If you will need more time than this to complete your revisions, please reply to this message or contact the journal office at <a href="mailto:plosone@plos.org">plosone@plos.org. When you're ready to submit your revision, log on to https://www.editorialmanager.com/pone/ and select the 'Submissions Needing Revision' folder to locate your manuscript file.

Please include the following items when submitting your revised manuscript:

- A rebuttal letter that responds to each point raised by the academic editor and reviewer(s). You should upload this letter as a separate file labeled 'Response to Reviewers'.
- A marked-up copy of your manuscript that highlights changes made to the original version. You should upload this as a separate file labeled 'Revised Manuscript with Track Changes'.
- An unmarked version of your revised paper without tracked changes. You should upload this as a separate file labeled 'Manuscript'.

Response: Thank you for your kind guidance. We have resubmitted our revised manuscript as requested.

If you would like to make changes to your financial disclosure, please include your updated statement in your cover letter. Guidelines for resubmitting your figure files are available below the reviewer comments at the end of this letter.

Response: Thank you for your helpful advice. We would like to change our financial disclosure and have updated the statement in the cover letter, while also resubmitting the image files in accordance with the guidelines.

If applicable, we recommend that you deposit your laboratory protocols in protocols.io to enhance the reproducibility of your results. Protocols.io assigns your protocol its own identifier (DOI) so that it can be cited independently in the future. For instructions see: https://journals.plos.org/plosone/s/submission-guidelines#loc-laboratory-protocols. Additionally, PLOS ONE offers an option for publishing peer-reviewed Lab Protocol articles, which describe protocols hosted on protocols.io. Read more information on sharing protocols at https://plos.org/protocols?utm_medium=editorial-email&utm_source=authorletters&utm_campaign=protocols.

We look forward to receiving your revised manuscript.

Kind regards,

Journal Requirements

When submitting your revision, we need you to address these additional requirements.

1. Please ensure that your manuscript meets PLOS ONE's style requirements, including those for file naming. The PLOS ONE style templates can be found at

https://journals.plos.org/plosone/s/file?id=wjVg/PLOSOne_formatting_sample_main_body.pdf and

https://journals.plos.org/plosone/s/file?id=ba62/PLOSOne_formatting_sample_title_authors_affiliations.pdf

Response: Thank you for your kind guidance. We have reformatted the manuscript according to the above style guidelines.

2. Please note that PLOS ONE has specific guidelines on code sharing for submissions in which author-generated code underpins the findings in the manuscript. In these cases, all author-generated code must be made available without restrictions upon publication of the work. Please review our guidelines at https://journals.plos.org/plosone/s/materials-and-software-sharing#loc-sharing-code and ensure that your code is shared in a way that follows best practice and facilitates reproducibility and reuse.

Response: Thank you for your kind guidance. We have shared our code in the Figshare database. Doi: https://doi.org/10.6084/m9.figshare.25335076.v1; https://doi.org/10.6084/m9.figshare.26197928.v1

3. Thank you for stating the following financial disclosure: "This work was supported in part by the Shandong Provincial Department of Science and Technology Research Project on Key Technologies for the Development of All-Terrain Intelligent Orchard Platform. (2019GNC106032)."
Please state what role the funders took in the study. If the funders had no role, please state: ""The funders had no role in study design, data collection and analysis, decision to publish, or preparation of the manuscript.""
If this statement is not correct you must amend it as needed.
Please include this amended Role of Funder statement in your cover letter; we will change the online submission form on your behalf.

Response: Thank you for your kind guidance. We have already provided a clarification in the cover letter regarding the role of the funders in the study.

Additional Editor Comments:

As per the reviewers recommendation, I am suggesting "Major Revision". Authors need to go through each comments of all the Reviewers and make a point to point correction and upgrade the paper to be eligible for further consideration in "PLOS ONE".

Response: Thank you for your advice. We will address reviewers' comments in the following pages.

Thank you again for your kind and helpful advice

AUTHOR'S RESPONSE TO REVIEWERS' COMMENTS

We greatly appreciate the reviewers taking the time to provide constructive comments and helpful suggestions. There is no doubt that the suggestions have significantly raised the quality if the manuscript and have enable us to improve the manuscript. Each suggested revision and comment brought forward by the reviewers was accurately incorporated and considered. We have carefully addressed all the reviewer's concerns. Please see our replies. Changes highlighted in red have been made accordingly in the revised manuscript.

Comments to the Author

---

1. Is the manuscript technically sound, and do the data support the conclusions?

The manuscript must describe a technically sound piece of scientific research with data that supports the conclusions. Experiments must have been conducted rigorously, with appropriate controls, replication, and sample sizes. The conclusions must be drawn appropriately based on the data presented.

Reviewer #1: Partly

Reviewer #2: Partly

Reviewer #3: Partly

Reviewer #4: Partly

Response: Thanks for your review. We have further improved the contrast experiments, and finally verified the performance of the improved YOLOv5 algorithm in bearing defect detection through ablation experiments and contrast experiments.

---

2. Has the statistical analysis been performed appropriately and rigorously?

Reviewer #1: Yes

Reviewer #2: No

Reviewer #3: No

Reviewer #4: No

Response: Thank you for agreeing that our analysis is appropriate and rigorous.We will further improve the statistical analysis.

---

3. Have the authors made all data underlying the findings in their manuscript fully available?

The PLOS Data policy requires authors to make all data underlying the findings described in their manuscript fully available without restriction, with rare exception (please refer to the Data Availability Statement in the manuscript PDF file). The data should be provided as part of the manuscript or its supporting information, or deposited to a public repository. For example, in addition to summary statistics, the data points behind means, medians and variance measures should be available. If there are restrictions on publicly sharing data—e.g. participant privacy or use of data from a third party—those must be specified.

Reviewer #1: Yes

Reviewer #2: Yes

Reviewer #3: No

Reviewer #4: Yes

Response: Thank you for your approval for our data.

4. Is the manuscript presented in an intelligible fashion and written in standard English?

PLOS ONE does not copyedit accepted manuscripts, so the language in submitted articles must be clear, correct, and unambiguous. Any typographical or grammatical errors should be corrected at revision, so please note any specific errors here.

Reviewer #1: Yes

Reviewer #2: No

Reviewer #3: No

Reviewer #4: Yes

Response: Thank you for your approval. We will carefully revise the manuscript for language quality.

Review Comments to the Author

Please use the space provided to explain your answers to the questions above. You may also include additional comments for the author, including concerns about dual publication, research ethics, or publication ethics. (Please upload your review as an attachment if it exceeds 20,000 characters)

Reviewer #1: A method of bearing defect detection based on an improved YOLOv5 algorithm is proposed to address the problem of low efficiency and easy omission in manual detection. The following problems are suggested to be improved.

1. There are problems in grammar which need to be improved.

Response: Thanks to the reviewer for pointing out the problem. We have improved the grammatical issues in the paper.

2. This manuscript only compares with YOLOv5s. The proposed algorithm should compared with YOLOv7 and YOLOv8.

Response: Thanks to the reviewer for pointing out the problem. We have augmented the "Comparison experiment" section by including a comparative analysis with other mainstream defect detection algorithms in terms of Precision (P), mean Average Precision (mAP), and Frames Per Second (FPS). Including a comparison with YOLOv7 and YOLOv8 algorithms.

Reviewer #2: The paper is based on the YOLOv5 object detection algorithm, and achieves partial performance improvement by incorporating the Res2Block module and a Bidirectional Feature Pyramid Network. However, there are still some shortcomings in the content of the paper. My suggestions are as follows:

1. What does "all mAP" in the abstract mean? Does it refer to the average mAP across the dataset, or is it the mAP improvement for each defect class? mAP is a metric with various types; which type are you referring to? The contribution section in the introduction has the same issue.

Response: Thanks to the reviewer for pointing out the problem. In the abstract and introduction sections of the paper, "all mAP" indeed refers to the average mAP across the dataset. We clarified this in the revised manuscript using red font.

2. The English writing needs improvement. For example, in the introduction, the sentence “Firstly, they enhanced the contrast of the images used, then applied the Hough algorithm to extract special

areas, and finally performed edge template matching on the images to determine if there are defects". Consider thoroughly revising the manuscript for language quality.

Response: Thanks to the reviewer for pointing out the problem. We have revised the relevant sentence content and retranslated the manuscript to improve the language quality.

---

3. When convolutional neural network is first mentioned in the introduction, it should be followed by its abbreviation (CNN) in parentheses; subsequently, only the abbreviation should be used.

Response: Thanks to the reviewer for pointing out the problem. We have revised the relevant content in the "Introduction" chapter and highlighted it with red font in the manuscript.

---

4. The first letter of a new sentence should be capitalized. Similar writing errors should be minimized throughout the manuscript.

Response: Thanks to the reviewer for pointing out the problem. We have revised the relevant content and highlighted it with red font in the manuscript.

---

5. In referencing Chinese authors, the surname should be used instead of the full name, as seen in references 2 and 14 in the introduction.

Response: Thanks to the reviewer for pointing out the problem. The following example is the official reference format for Chinese authors in PLOS ONE, and we have modified the corresponding references in the manuscript according to this format and highlighted them in red font .

Example： Hou WR, Hou YL, Wu GF, Song Y, Su XL, Sun B, et al. cDNA, genomic sequence cloning and over expression of ribosomal protein gene L9 (rpL9) of the giant panda (Ailuropoda melanoleuca). Genet Mol Res. 2011;10: 1576-1588.

---

6. Defect names in the dataset should not be in Pinyin, such as "cashang, huahen, aocao." This applies to the tables of experimental results as well.

Response: Thanks to the reviewer for pointing out the problem. We have provided explanations for the names of relevant bearing defects and have marked the relevant content in red font in the "Dataset" section, "Model performance" section, "Ablation experiment" section and "Comparative experiment" section of the manuscript.

---

7. Comparing training and validation losses separately is not necessary. Consider using a table to compare the training losses between different networks.

Response: Thanks you for your advice. After carefully considering your suggestions, we realize that while using a table to compare the training losses of different networks is concise and clear, it may not visually illustrate the changing trend of the losses during the training process. Therefore, we have decided to continue using our original approach to display the loss changes of different models during training. Meanwhile, to maintain the conciseness and focus of the paper, we have decided to only retain the comparison of training losses and omit the comparison of data loss curves during the validation process.

---

8. When comparing model performance, specify which defect class achieves the stated mAP value, as in "the YOLOv5 algorithm achieves an mAP50 value of 0.939 when detecting bearing defects."

Response: Thanks to the reviewer for pointing out the problem. We have made revisions to the relevant content in the "Model performance" section and highlighted the related content in red font

in the manuscript.

9. Consider merging Tables 1 and 2 into a single table. The term "All" in the table needs to be clearly defined in the manuscript.

Response: Thanks you for your advice. We have merged Table 1 and Table 2 into a new Table 1. The corresponding content in the "Model performance" section of the manuscript has been marked in red. In the table, "All" refers to the average mAP across the dataset.

10. Besides the proposed algorithm, there is only one comparative algorithm mentioned. It might be beneficial to include additional algorithms for comparison, such as YOLOv3, which you mentioned in the paper, to further validate the advantages of your proposed method in terms of inference speed and performance.

Response: Thanks to the reviewer for pointing out the problem. We have augmented the "Comparison experiment" section by including a comparative analysis with other mainstream defect detection algorithms in terms of Precision (P), mean Average Precision (mAP), and Frames Per Second (FPS). Including a comparison with YOLOv3 algorithms.

11. In the ablation study, clarify what "YOLOv5s+All" means. Ensure that this is clearly described in the manuscript and that the figures (or tables) and text are consistent.

Response: Thanks to the reviewer for pointing out the problem. "YOLOv5s+All" means "YOLOv5s+BiFPN+Res2Block". We have provided an explanation for the relevant content in the "Ablation experiment" section, and highlighted it in red font in the manuscript.

Reviewer #3: To address the issues of low efficiency and easy omission of manual detection, the authors proposed an improved YOLOv5 algorithm to detect the bearing defect. After reading the whole paper, I think the contribution is weak, see the detailed comments as follows:

1. The innovation points of this article are general, where the proposed method is simply modified through YOLOv5.

Response: Thanks to the reviewer for pointing out the problem. Although it may seem like we have merely modified the YOLOv5 algorithm superficially, in reality, the modifications were targeted and deliberate. We optimized and improved several crucial parts of YOLOv5 specifically addressing the unique challenges and difficulties of bearing defect detection tasks. For instance, we refined the network architecture to enhance the detection capability for minute bearing defects; optimized the feature extraction ability, allowing it to capture finer details in images and thus improving the recognition of small target defects in bearings; and enhanced the feature fusion capacity, effectively integrating shallow image features with deep semantic features, resulting in a reduction in missed detections and an increase in the accuracy of bearing defect recognition. These improvements are not mere superficial modifications; they have been thoroughly researched and experimentally validated.

To validate the effectiveness of our approach, we conducted experiments on dataset and compared it with the original YOLOv5 and other relevant defect detection methods. The results demonstrated that our method achieved superior performance in bearing defect detection tasks, not only improving accuracy but also exhibiting robustness in scenarios with dense and overlapping targets. These experimental outcomes strongly confirm that our method is not just a simple modification of YOLOv5 but possesses both practical significance and utility.

Based on your valuable suggestions, we will further delve into the research and exploration of new methods and technologies for bearing defect detection. We will continue to keep abreast of the latest research achievements and technological advancements in this field, and strive to incorporate more innovative elements into our research.

2. Currently, the YOLO series has developed up to YOLOv10. Why is the author still using YOLOv5?

Response: Thanks to the reviewer for pointing out the problem. The research background of this paper is bearing defect detection. Considering our experimental conditions, the characteristics of the dataset, and the availability of computational resources, we chose to improve upon the YOLOv5 algorithm instead of directly applying YOLOv10.

Since its release, YOLOv5 has been widely used and validated in various fields, demonstrating high stability and maturity in its algorithm. This is crucial for both our experimental research and practical applications. Although YOLOv5 has already demonstrated excellent performance in certain aspects, our team have made improvements based on YOLOv5., which have significantly enhanced the algorithm's performance in bearing defect detection tasks. Additionally, we have conducted comparative experiments and analyses with YOLOv3, YOLOv6, YOLOv7, and YOLOv8 to further validate the detection capabilities of our improved model.

We fully recognize the performance improvements of YOLOv10 and remain actively engaged in following its latest research progress. In our future work, we plan to introduce YOLOv10 into our research and conduct a more in-depth comparison and analysis with YOLOv5.

3. The literature review should be improved with the latest studies, and the research gap should be thoroughly investigated.

Response: Thanks to the reviewer for pointing out the problem. I agree with your emphasis on the importance of incorporating more recent studies into the literature review and thoroughly exploring research gaps.

I focused on research outcomes within a specific time period primarily because these studies are closely related to and effectively support my research hypotheses and framework. Concurrently, due to limitations in time and resources, it was challenging to comprehensively track and evaluate the latest published research. Consequently, in response to your invaluable suggestions, I will, in future research endeavors, continuously monitor and incorporate the latest research findings, while also conducting a thorough analysis of research gaps to further consolidate and expand my research directions.

4. It should be clearly stated what the past researchers did, what their limitations were, and how the current study adds new knowledge to the existing literature.

Response: Thanks to the reviewer for pointing out the problem. In the introduction, we clearly pointed out that previous bearing defect detection methods often suffered from low detection accuracy when dealing with complex scenarios such as overlapping and occlusion of bearings, as well as dense occurrences of multiple defects and small target defects. To address these issues, this paper conducted in-depth research and proposed an improved detection model. As evidenced by the detection results, our model has demonstrated good detection performance in these complex scenarios, effectively improving the recognition accuracy of bearing defects.

5. The experimental section lacks comparative experiments with other state-of-the-art models, which makes it difficult to validate the effectiveness of the proposed method.

Response: Thanks to the reviewer for pointing out the problem. we have conducted comparative experiments and analyses with YOLOv3, YOLOv6, YOLOv7, and YOLOv8 to further validate the detection capabilities of our improved model.

---

6. Language expression needs to be improved.

Response: Thanks to the reviewer for pointing out the problem. We have retranslated the manuscript to improve the language quality.

Reviewer #4: Overall the innovation and workload of this paper are insufficient in some degrees. It is expected that the author will carefully revise the suggestions provided in the future, improve the details of the experiment, supplement the comparative experiment, and reconsider the overall framework of the paper. It is also suggested to discuss more with other co-authors before submitting the revised draft to improve the quality of the paper. Below are some technical comments.

1. The overall format of the paper should be aligned at both ends, but the text format does align to the left.

Response: Thanks to the reviewer for pointing out the problem. We have set the content of the manuscript to be aligned at both ends.

---

2. Abstract part lacks the keyword extraction and writing.

Response: Thanks to the reviewer for pointing out the problem. While writing this paper, we utilized the LaTeX standard template provided by the journal and referenced the formatting styles of papers published in the journal over the past two years. The keywords are not included within the text but were submitted as a separate section. The keywords for this paper are: YOLOv5; Bearing Defects; Feature Extraction; Feature Fusion; Object Detection.

---

3. In the paper review, some researchers have achieved good experimental results in the detection of bearing defects, and the detection result is as high as 98.6%. Therefore, they have doubts about the author's purpose of selecting the topic and carrying out the research. At the same time, the author sorts out the review and points out that some scholars' research methods have certain limitations, and these problems are not fully solved in the following paper.

Response: Thanks to the reviewer for pointing out the problem. I am well aware that researchers have achieved a detection accuracy of 98.6% in bearing defect detection. These research achievements provide valuable references and insights for our work.

We have noticed that although Chen Shuo's method of using the end face of bearing rings to detect bearing defects achieved a detection accuracy of 98.6%, its recognition accuracy and robustness are insufficient in complex scenarios involving overlapping and occlusion among bearings. The research presented in this paper aims to address and improve upon these limitations.

Firstly, the bearing dataset used in this paper was collected under dense target and overlapping target scenarios, and data augmentation techniques were employed to further enrich the diversity of the dataset. Secondly, we conducted a series of targeted improvements to the YOLOv5 algorithm, including enhancements in feature extraction and feature fusion. As can be seen from the subsequent

detection results, the improved algorithm presented in this paper demonstrates good accuracy in both dense target and overlapping target scenarios.

Therefore, despite the high detection accuracy achieved by previous studies, our research still holds significant importance and value.

---

4. The size of all the pictures in the paper is too large and in low resolution. For instance, in Fig1 & Fig2, the images are blurry and you can't even see the words in the red background. Please ask the authors to improve the clarity of the images and consider adjusting the font or color of the text to make the images legible.

Response: Thanks to the reviewer for pointing out the problem. Regarding the issue you mentioned about the blurriness of images in Figures 1 and 2, as well as the illegibility of text against the red background, we have made adjustments. Additionally, we have also replaced other problematic images with clearer ones. We appreciate your feedback and strive to ensure the clarity and readability of our materials.

---

5. Both Res2Block and BiFPN modules were proposed in 2019, and this year is 2024. Whether the improvement strategy has fallen behind, it is suggested that the author should contact or study some cutting-edge articles in the field of computer vision to ensure that the research process keeps pace with The Times.

Response: Thanks you for your advice. We fully understand your concern about Res2Block and BiFPN modules as relatively early technologies, and we agree that maintaining technological advancement is crucial in the rapidly developing field of computer vision.

In this paper, we have leveraged the residual learning mechanism of Res2Block to optimize the network structure of YOLOv5, enhancing the model's feature extraction and representation capabilities. Meanwhile, we have borrowed the feature fusion strategy of BiFPN to improve the feature fusion method of YOLOv5, enabling the model to better adapt to the task of bearing defect detection. The effectiveness of our approach has been demonstrated through subsequent Detetion result and ablation experiment

Based on your valuable suggestions, we will further delve into the research and exploration of new methods and technologies for bearing defect detection. We will continue to keep abreast of the latest research achievements and technological advancements in this field, and strive to incorporate more innovative elements into our research.

---

6. The Neck part of the original YOLOv5 model adopted the feature pyramid (FPN) and path aggregation network (PANet) structures, while the author only described the PANet structure in the relevant chapters of the paper and used BiFPN module to replace the PANet structure, which indicates that the author has not a comprehensive understanding of the YOLOv5 network structure. It is suggested that the author revise and study the relevant articles carefully.

Response: Thanks to the reviewer for pointing out the problem. We have made modifications to the relevant content in the "Overall architecture of the model" section, and highlighted it with red font in the manuscript.

---

7. When introducing the dataset, the author has introduced the enhancement method of the dataset in detail. There are overlapping parts in this part, so it is suggested that the author delete the relevant chapters.

Response: Thanks you for your advice. We have deleted the content about data augmentation methods in the "Dataset" section.

8. The author didn't introduce the setting of relevant experimental parameters, so it is suggested that the author add this part.

Response: Thanks you for your advice. We have included the "Relevant experimental parameters" section in the "Pre-experiment preparation" chapter, and have set the font to red in the manuscript.

9. The index of detection rate FPS is not given the full name in English and calculation formula.

Response: Thanks to the reviewer for pointing out the problem. We have added the full name and calculation formula of FPS in the "Experimental evaluation indicators" section, and highlighted them in red font in the manuscript.

10. The author should explicitly use the model of YOLOv5 version in the whole paper, such as YOLOv5s, YOLOv5m, YOLOv5l and YOLOv5x.

Response: Thanks to the reviewer for pointing out the problem. We have clarified in the "Overall architecture of the model" section that this paper employs the YOLOv5s version of the YOLOv5 algorithm, and have highlighted this information in red font in the manuscript.

11. What is Small obj. algorithm? The improved algorithm is not compared with the original YOLOv5s algorithm in detail, but only provides the improved results of various indicators. It is suggested to supplement the experimental results of YOLOv5s in Table 1 and Table 2.

Response: Thanks you for your advice. The Small_obj algorithm is an existing method that involves adding a small target detection head for the purpose of identifying small target defects. Figure 1 illustrates the structural model of this algorithm.

In the "Model Performance" section, we have merged Table 1 and Table 2, and added comparisons with the original YOLOv5s algorithm in terms of accuracy, recall, mAP, and FPS.

12. It is suggested that the author adjust the relevant chapters of comparison experiment and ablation experiment to ensure the overall logic of the article.

Response: Thanks you for your advice. We have adjusted the "Comparison experiment" and "Ablation experiment" section.

13. There are too few algorithms in the comparative experiment to demonstrate the superiority of the improved YOLOv5s algorithm in this paper. At the same time, in the literature review section, a scholar's detection result reached 98.6%, while the mAP of the algorithm proposed in this paper is 88.4%. We hope the author can provide stronger evidence to prove the superiority of the algorithm in this paper.

Response: Thanks to the reviewer for pointing out the problem. We have augmented the "Comparison experiment" section by including a comparative analysis with other mainstream defect detection algorithms in terms of Precision (P), mean Average Precision (mAP), and Frames Per Second (FPS).

I am well aware that researchers have achieved a detection accuracy of 98.6% in bearing defect detection. These research achievements provide valuable references and insights for our work.

We have noticed that although Chen Shuo's method of using the end face of bearing rings to detect bearing defects achieved a detection accuracy of 98.6%, its recognition accuracy and robustness are

insufficient in complex scenarios involving overlapping and occlusion among bearings. The research presented in this paper aims to address and improve upon these limitations.

Firstly, the bearing dataset used in this paper was collected under dense target and overlapping target scenarios, and data augmentation techniques were employed to further enrich the diversity of the dataset. Secondly, we conducted a series of targeted improvements to the YOLOv5 algorithm, including enhancements in feature extraction and feature fusion. As can be seen from the subsequent detection results, the improved algorithm presented in this paper demonstrates good accuracy in both dense target and overlapping target scenarios.

Therefore, despite the high detection accuracy achieved by previous studies, our research still holds significant importance and value.

---

14. In Table1, Table 2 and Table 3, the names of the defects in the tables are in Chinese Pinyin, please replace the Chinese names with the corresponding English ones to avoid doubts from non-Chinese speakers. If necessary, explain the detected parts in the text in the form of words or pictures. For example, 'cashang' means 'xxx (English Word)'

Response: Thanks to the reviewer for pointing out the problem.We have adjusted the relevant content in the sections of "Dataset", "Model performance", "Ablation experiment", and "Comparative experiment". And highlighted them in red font in the manuscript.

---

15. It is better to add more textual information about the type of bearing damage in Fig 5

Response: Thanks you for your advice. We have added text explanations for the defect types in Figure 5.

---

6. PLOS authors have the option to publish the peer review history of their article (what does this mean?). If published, this will include your full peer review and any attached files.

If you choose "no", your identity will remain anonymous but your review may still be made public.

Do you want your identity to be public for this peer review? For information about this choice, including consent withdrawal, please see our Privacy Policy.
Reviewer #1: No
Reviewer #2: No
Reviewer #3: No
Reviewer #4: No

[NOTE: If reviewer comments were submitted as an attachment file, they will be attached to this email and accessible via the submission site. Please log into your account, locate the manuscript record, and check for the action link "View Attachments". If this link does not appear, there are no attachment files.]

While revising your submission, please upload your figure files to the Preflight Analysis and Conversion Engine (PACE) digital diagnostic tool, https://pacev2.apexcovantage.com/. PACE helps ensure that figures meet PLOS requirements. To use PACE, you must first register as a user. Registration is free. Then, login and navigate to the UPLOAD tab, where you will find detailed instructions on how to use the tool. If you encounter any issues or have any questions when using PACE, please email PLOS at <a

href="mailto:figures@plos.org">figures@plos.org. Please note that Supporting Information files do not need this step.

In compliance with data protection regulations, you may request that we remove your personal registration details at any time. (Remove my information/details). Please contact the publication office if you have any questions.
