# Supplementary material for: Bearing defect detection based on the improved YOLOv5 algorithm
Source: PLoS One. 2024 Oct 28;19(10):e0310007. doi: 10.1371/journal.pone.0310007 (PMC11515976; doi:10.1371/journal.pone.0310007)
Supplement: Attachment — Submitted filename: Response to Reviewers.pdf [file pone.0310007.s002.pdf]

AUTHORS'RESPONSE TO EDITORS AND REVIEWERS

Dear Editors and Reviewers,

Thank you very much for your kindly comments on our manuscript. There is no doubt that these comments are valuable and very helpful for revising and improving our manuscript. We are pleased to submit the revised research article “Bearing defect detection based on the improved YOLOv5 algorithm”. On the following pages, we present our point-by-point responses to the comments.

We would like also to thank you for allowing us to resubmit a revised copy of the manuscript. We appreciate your time and look forward to your response.

Sincerely,
Peigang Jiao,Ph.D. (corresponding author)
jiaopeigang@163.com

Comments from Academic Editor

Thank you for submitting your manuscript to PLOS ONE. After careful consideration, we feel that it has merit but does not fully meet PLOS ONE ' s publication criteria as it currently stands. Therefore, we invite you to submit a revised version of the manuscript that addresses the points raised during the review process.

Dear authors, we have now received reviewers feedback regarding your revised paper. As per their feedback, authors have upgrade and improved the paper content based on the suggestions provided. However, one reviewer is not convinced regarding the innovation in this article and the reviewer thinks it is not sufficient. Another reviewer suggest, the author should add relevant comparative experiments to compare the current mainstream object detection algorithms to verify the superiority of the improved algorithm in the comparative experiment section and more convincing explanations need to be added. In view of their comments, I am recommending "Minor Revision" to address this flaws. Authors are requested to go through the comments and made sufficient improvement of their paper.

Please submit your revised manuscript by Sep 06 2024 11:59PM. If you will need more time than this to complete your revisions, please reply to this message or contact the journal office at <a href="mailto:plosone@plos.org">plosone@plos.org. When you're ready to submit your revision, log on to https://www.editorialmanager.com/pone/ and select the 'Submissions Needing Revision' folder to locate your manuscript file.

Please include the following items when submitting your revised manuscript:

- A rebuttal letter that responds to each point raised by the academic editor and reviewer(s). You should upload this letter as a separate file labeled 'Response to Reviewers'.
- A marked-up copy of your manuscript that highlights changes made to the original version. You should upload this as a separate file labeled 'Revised Manuscript with Track Changes'.
- An unmarked version of your revised paper without tracked changes. You should upload this as a separate file labeled 'Manuscript'.

Response: Thank you for your kind guidance. We have resubmitted our revised manuscript as requested.

If you would like to make changes to your financial disclosure, please include your updated statement in your cover letter. Guidelines for resubmitting your figure files are available below the reviewer comments at the end of this letter.

Response: Thank you for your helpful advice. We don't need to revise the financial disclosure.

If applicable, we recommend that you deposit your laboratory protocols in protocols.io to enhance the reproducibility of your results. Protocols.io assigns your protocol its own identifier (DOI) so that it can be cited independently in the future. For instructions see:

https://journals.plos.org/plosone/s/submission-guidelines#loc-laboratory-protocols. Additionally, PLOS ONE offers an option for publishing peer-reviewed Lab Protocol articles, which describe protocols hosted on protocols.io. Read more information on sharing protocols at https://plos.org/protocols?

utm_medium=editorial-email&utm_source=authorletters&utm_campaign=protocols.

We look forward to receiving your revised manuscript.

Kind regards,

Journal Requirements

Please review your reference list to ensure that it is complete and correct. If you have cited papers that have been retracted, please include the rationale for doing so in the manuscript text, or remove these references and replace them with relevant current references. Any changes to the reference list should be mentioned in the rebuttal letter that accompanies your revised manuscript. If you need to cite a retracted article, indicate the article' s retracted status in the References list and also include a citation and full reference for the retraction notice.

Response: Thank you for your kind guidance. We have checked the reference list to ensure that it is complete and correct.

Additional Editor Comments:

Dear authors, we have now received reviewers feedback regarding your revised paper. As per their feedback, authors have upgrade and improved the paper content based on the suggestions provided. However, one reviewer is not convinced regarding the innovation in this article and the reviewer thinks it is not sufficient. Another reviewer suggest, the author should add relevant comparative experiments to compare the current mainstream object detection algorithms to verify the superiority of the improved algorithm in the comparative experiment section and more convincing explanations need to be added. In view of their comments, I am recommending "Minor Revision" to address this flaws. Authors are requested to go through the comments and made sufficient improvement of their paper.

Response: Thank you for your advice. We will address reviewers' comments in the following pages.

AUTHOR'S RESPONSE TO REVIEWERS' COMMENTS

We greatly appreciate the reviewers taking the time to provide constructive comments and helpful suggestions. There is no doubt that the suggestions have significantly raised the quality if the manuscript and have enable us to improve the manuscript. Each suggested revision and comment brought forward by the reviewers was accurately incorporated and considered. We have carefully addressed all the reviewer's concerns. Please see our replies. Changes highlighted in red have been made accordingly in the revised manuscript.

Comments to the Author

1. If the authors have adequately addressed your comments raised in a previous round of review and you feel that this manuscript is now acceptable for publication, you may indicate that here to bypass the "Comments to the Author" section, enter your conflict of interest statement in the "Confidential to Editor" section, and submit your "Accept" recommendation.

Reviewer #1: All comments have been addressed

Reviewer #2: (No Response)

Reviewer #3: All comments have been addressed

Reviewer #4: All comments have been addressed

Response: Thank you for your approval.

2. Is the manuscript technically sound, and do the data support the conclusions?

The manuscript must describe a technically sound piece of scientific research with data that supports the conclusions. Experiments must have been conducted rigorously, with appropriate controls, replication, and sample sizes. The conclusions must be drawn appropriately based on the data presented.

Reviewer #1: Yes

Reviewer #2: Partly

Reviewer #3: Yes

Reviewer #4: Partly

Response: Thanks for your review. We have further improved the contrast experiments, and finally verified the performance of the improved YOLOv5 algorithm in bearing defect detection through ablation experiments and contrast experiments.

3. Has the statistical analysis been performed appropriately and rigorously?

Reviewer #1: Yes

Reviewer #2: Yes

Reviewer #3: Yes

Reviewer #4: Yes

Response: Thank you for agreeing that our analysis is appropriate and rigorous.

4. Have the authors made all data underlying the findings in their manuscript fully available?

The PLOS Data policy requires authors to make all data underlying the findings described in their manuscript fully available without restriction, with rare exception (please refer to the Data Availability Statement in the manuscript PDF file). The data should be provided as part of the manuscript or its supporting information, or deposited to a public repository. For example, in addition to summary

statistics, the data points behind means, medians and variance measures should be available. If there are restrictions on publicly sharing data—e.g. participant privacy or use of data from a third party—those must be specified.

Reviewer #1: Yes

Reviewer #2: Yes

Reviewer #3: Yes

Reviewer #4: Yes

Response: Thank you for your approval for our data.

---

5. Is the manuscript presented in an intelligible fashion and written in standard English?

PLOS ONE does not copyedit accepted manuscripts, so the language in submitted articles must be clear, correct, and unambiguous. Any typographical or grammatical errors should be corrected at revision, so please note any specific errors here.

Reviewer #1: Yes

Reviewer #2: No

Reviewer #3: Yes

Reviewer #4: Yes

Response: Thank you for your approval. We will carefully revise the manuscript for language quality.

Review Comments to the Author

Please use the space provided to explain your answers to the questions above. You may also include additional comments for the author, including concerns about dual publication, research ethics, or publication ethics. (Please upload your review as an attachment if it exceeds 20,000 characters)

Reviewer #1: (No Response)

Reviewer #2: The author has made some improvements to the content based on the suggestions provided. However, the innovation in this article is not sufficient.

Response: Thanks to the reviewer for pointing out the problem. We modifications to the YOLOv5 algorithm are targeted and well-considered. We optimized and improved several crucial parts of YOLOv5 specifically addressing the unique challenges and difficulties of bearing defect detection tasks. For instance, we refined the network architecture to enhance the detection capability for minute bearing defects; optimized the feature extraction ability, allowing it to capture finer details in images and thus improving the recognition of small target defects in bearings; and enhanced the feature fusion capacity, effectively integrating shallow image features with deep semantic features, resulting in a reduction in missed detections and an increase in the accuracy of bearing defect recognition. These improvements are not mere superficial modifications; they have been thoroughly researched and experimentally validated.

To validate the effectiveness of our approach, we conducted experiments on dataset and compared it with the original YOLOv5 and other relevant defect detection methods. The results demonstrated that our method achieved superior performance in bearing defect detection tasks, not only improving accuracy but also exhibiting robustness in scenarios with dense and overlapping targets. These

experimental outcomes strongly confirm that our method is not just a simple modification of YOLOv5 but possesses both practical significance and innovation.

Based on your valuable suggestions, we will further delve into the research and exploration of new methods and technologies for bearing defect detection. We will continue to keep abreast of the latest research achievements and technological advancements in this field, and strive to incorporate more innovative elements into our research.

Reviewer #3: (No Response)

Reviewer #4: After careful modification by the author, the overall quality of this paper has been further improved. The topic selection of this paper has certain research significance, the structure and logic of the paper are clear, the language expression is standardized, and the research results have certain construction significance.
Finally, it is suggested that the author try to pay attention to and contact with some current mainstream and newly launched object detection algorithms. Improve the comparison experiment part, improve the persuasiveness and superiority of the improved algorithm proposed in this paper.

Response: Thanks you for your advice. We have improved the comparison experiment part and highlighted it in red in the manuscript.

---

7. PLOS authors have the option to publish the peer review history of their article (what does this mean?). If published, this will include your full peer review and any attached files.

If you choose “no”, your identity will remain anonymous but your review may still be made public.

Do you want your identity to be public for this peer review? For information about this choice, including consent withdrawal, please see our Privacy Policy.
Reviewer #1: No
Reviewer #2: No
Reviewer #3: No
Reviewer #4: No
[NOTE: If reviewer comments were submitted as an attachment file, they will be attached to this email and accessible via the submission site. Please log into your account, locate the manuscript record, and check for the action link "View Attachments". If this link does not appear, there are no attachment files.]

While revising your submission, please upload your figure files to the Preflight Analysis and Conversion Engine (PACE) digital diagnostic tool, https://pacev2.apexcovantage.com/. PACE helps ensure that figures meet PLOS requirements. To use PACE, you must first register as a user. Registration is free. Then, login and navigate to the UPLOAD tab, where you will find detailed instructions on how to use the tool. If you encounter any issues or have any questions when using PACE, please email PLOS at <a href="mailto:figures@plos.org">figures@plos.org. Please note that Supporting Information files do not need this step.

In compliance with data protection regulations, you may request that we remove your personal registration details at any time. (Remove my information/details). Please contact the publication office if you have any questions.
